# Manual on the proper use of lutetium-177-labeled somatostatin analogue (Lu-177-DOTA-TATE) injectable in radionuclide therapy (2nd ed.)

**DOI:** 10.1007/s12149-018-1230-7

**Published:** 2018-01-15

**Authors:** Makoto Hosono, Hideharu Ikebuchi, Yoshihide Nakamura, Nobutaka Nakamura, Takahiro Yamada, Sachiko Yanagida, Asami Kitaoka, Kiyotaka Kojima, Hiroyasu Sugano, Seigo Kinuya, Tomio Inoue, Jun Hatazawa

**Affiliations:** 1 Faculty of Medicine, Kindai University, 377-2 Ohno-Higashi, Osaka-Sayama, Osaka 589-8511 Japan; 2 Japanese Society of Nuclear Medicine, 2-28-45 Honkomagome, Bunkyo-ku, Tokyo, 113-0021 Japan; 3 Japan Radioisotope Association, 2-28-45 Honkomagome, Bunkyo-ku, Tokyo, 113-0021 Japan; 4 FUJIFILM RI Pharma Co., Ltd., 2-14-1 Kyobashi, Chuo-Ku, Tokyo, 104-0031 Japan

**Keywords:** Lu-177-DOTA-TATE, Radionuclide therapy, Neuroendocrine tumor

## Abstract

Here we present the guideline for the treatment of neuroendocrine tumors using Lu-177-DOTA-TATE on the basis of radiation safety aspects in Japan. This guideline was prepared by a study supported by Ministry of Health, Labour, and Welfare, and approved by Japanese Society of Nuclear Medicine. Lu-177-DOTA-TATE treatment in Japan should be carried out according to this guideline. Although this guideline is applied in Japan, the issues for radiation protection shown in this guideline are considered internationally useful as well. Only the original Japanese version is the formal document.

## Goals of radiation safety management

This Manual covers radiation safety management in conjunction with the use of a lutetium-177 (Lu-177)-labeled somatostatin analogue (Lu-177-DOTA-TATE) injectable (denoted here as “the labeled somatostatin analogue”) to treat an unresectable or metastatic neuroendocrine tumor (denoted here as “therapy with the labeled somatostatin analogue”), and this Manual was compiled to comply with principles of safety guidelines related to “Release of Patients who have been Administered a Radiopharmaceutical” (Notice No. 70 from the Safety Division, Pharmaceutical and Medical Safety Bureau dated June 30, 1998, denoted here as “Notice No. 70 from the Safety Division, Pharmaceutical and Medical Safety Bureau”) [1], which was superseded by “Release of Patients who have been Administered a Radiopharmaceutical” (Notice No. 1108-2 from the Division for Guidance on Medical Care, Health Policy Bureau dated November 8, 2010, denoted here as “Notice No. 1108-2 from the Division for Guidance on Medical Care, Health Policy Bureau”) [2] issued by the Ministry of Health, Labour, and Welfare. This Manual was also compiled to ensure the safe handling of the labeled somatostatin analogue.

Unresectable or metastatic neuroendocrine tumors often have a poor prognosis, and neuroendocrine tumors of the pancreas or gastrointestinal tract express high levels of somatostatin receptors. Phase I and II clinical trials of the labeled somatostatin analogue have recently been conducted overseas, and the results of those trials have indicated that the labeled somatostatin analogue has satisfactory antitumor action and improves QOL [3–5], as shown in Table 1.

**Table 1:** Clinical outcomes for patients administered Lu-177-DOTA-TATE

Authors	Number of patients	Complete response	Partial response	Complete response + partial response (%)
Sward et al. [3]	16	0 (0%)	6 (38%)	38
Kwekkeboom et al. [4]	310	5 (2%)	86 (28%)	30
Bodei et al. [5]	51	1 (2%)	14 (27%)	29

Nevertheless, the labeled somatostatin analogue must be administered at a dose of 7400 MBq × 4 (at an interval of about 8 weeks) [6] for the therapy to have a marked therapeutic benefit. Thus, medical personnel who perform the therapy must fully understand the physical and chemical properties of Lu-177.

Radionuclide therapy is a molecularly targeted therapy that administers a radioactive drug. This drug selectively collects in affected areas, such as groups of metastatic tumor cells throughout the patient’s body, and these areas are locally irradiated. For a patient to undergo radionuclide therapy that is minimally invasive, gentle, and safe, the labeled somatostatin analogue must be safely handled and measures must be taken to prevent exposure to radiation and prevent contamination. Thus, relevant parties such as patients and their family members must fully understand the characteristics of this form of internal radiotherapy with an RI.

This Manual incorporates the import of recommendations from the Medical Services Act and international agencies regarding protection from radiation [7–11], and so hospitals or other medical facilities should perform internal radiotherapy with the labeled somatostatin analogue in accordance with the requirements to ensure radiation safety that are covered in this Manual. Accordingly, the following points are summarized in this Manual on radiation safety management.

1. Guidelines for facility management
2. Protection from exposure
3. Storage and disposal of radioactive contaminants from medical sources

In addition, facilities performing therapy with the labeled somatostatin analogue need to meet the following criteria:

① A hospital or clinic performing the therapy (denoted here as “a hospital or other medical facility”) will meet criteria for radiological protection in medicine as stipulated in relevant laws and ordinances and follow all legal and regulatory procedures.
② Therapy with the labeled somatostatin analogue will be performed at a hospital or other medical facility employing physicians and radiology technologists with sufficient knowledge of and experience handling radiopharmaceuticals. In addition, the therapy will be performed at a hospital or other medical facility employing physicians with expertise and experience treating neuroendocrine tumors.
③ A hospital or other medical facility will receive accreditation from the Japanese Society of Nuclear Medicine (such a hospital or other medical facility is denoted here as an “accredited hospital or other medical facility”). Personnel at that hospital or other medical facility will then undergo training in radiation safety management of a specified duration before performing the therapy. An accredited hospital or other medical facility will employ at least one accredited physician and one accredited radiology technologist.

## Organizational efforts at a hospital or other medical facility performing therapy with the labeled somatostatin analogue

Given the peculiarities of the labeled somatostatin analogue, a hospital or other medical facility performing therapy with the labeled somatostatin analogue must satisfy the requirements of “Facilities at a hospital or other medical facility performing therapy with the labeled somatostatin analogue” to “Provisions to comply with when performing therapy with the labeled somatostatin analogue” in this section. This is to ensure that the therapy is provided by a team of medical personnel including physicians, radiology technologists managing radiation safety, and nurses providing patient care and assistance.

### Facilities at a hospital or other medical facility performing therapy with the labeled somatostatin analogue

Rooms where radiopharmaceuticals are used at a hospital or other medical facility performing the therapy will meet the criteria as stipulated in Article 30, Sections 8, 9, and 11 of the Ordinance for Enforcement of the Medical Services Act, and facilities will meet the criteria in Article 30, Section 13–26 of the Ordinance. A hospital or other medical facility will be approved by the governor of a prefecture or mayor of a municipality overseeing such facilities.

### Establishment of a safety management system at a hospital or other medical facility performing therapy with the labeled somatostatin analogue

To ensure medical safety, safe handling of the labeled somatostatin analogue, and radiation safety, the administrator of a hospital or other medical facility performing the therapy must have physicians who will perform the therapy and radiology technologists who will ensure radiation safety and medical safety participate in a workshop on radiation safety management [Workshop on Proper Use of an Lu-177-DOTA-TATE Injectable (tentative name) (denoted here as the “workshop on radiation safety and handling”)]. This workshop will be conducted at an accredited hospital or other medical facility for a specified period. In addition, the therapy will be performed within a system for “therapy with a Lu-177-DOTA-TATE injectable.” This system will be a part of the safety management system for coordinated medical safety at a hospital or other medical facility.

#### Designation and role of a radiation safety supervisor in relation to therapy with the labeled somatostatin analogue

The administrator of a hospital or other medical facility performing therapy with the labeled somatostatin analogue will designate a radiation safety supervisor for the therapy. The radiation safety supervisor will be an “accredited” physician who has gained expertise in the therapy through the workshop on radiation safety and handling. The radiation safety supervisor will supervise and oversee the therapy and the radiation safety supervisor will conduct training for relevant personnel such as physicians who will be performing the therapy.

#### Designation and role of a radiation safety officer in relation to therapy with the labeled somatostatin analogue

The administrator of a hospital or other medical facility performing therapy with the labeled somatostatin analogue will designate at least one radiation safety officer in accordance with conditions at the hospital or other medical facility. A radiation safety officer will be an “accredited” radiology technologist or nurse who has gained expertise in radiation safety management in relation to the therapy through the workshop on radiation safety and handling. Under the supervision of the radiation safety supervisor, a radiation safety officer will work to ensure radiation safety and manage radiation safety in relation to the therapy. A radiation safety officer will also conduct training in performing the therapy.

### Provisions to comply with when performing therapy with the labeled somatostatin analogue

This Manual lays out conditions for performing therapy with the labeled somatostatin analogue. The following provisions must be complied with.

1. The labeled somatostatin analogue will be administered to a patient with an unresectable or metastatic neuroendocrine tumor.
2. When an expert such as a radiation safety officer provides instructions such as precautions regarding the therapy to an eligible patient and his or her family members (or caregivers) prior to treatment, the expert will determine whether the patient can function normally in line with these instructions. The therapy will be performed if the patient and his or her family members (or caregivers) can follow and consent to follow these instructions.
3. After discharge, a patient’s residence will be equipped with appropriate sewage and a flush toilet.
4. The patient is able to function normally in terms of decision-making and behavior.
5. When a patient is discharged, contact between the patient and infants and pregnant women will be minimized.

## Characteristics of Lu-177 and the labeled somatostatin analogue

### Characteristics of Lu-177

The physical properties of the lutetium 177 (Lu-177) nuclide are as shown in Table 2 below.

**Table 2:** Physical properties of Lu-177

Half-life	Type of decay	Maximum energy (MeV) of β rays and percentage emitted	Photon energy (MeV) and percentage emitted	Percentage of internal conversion electrons emitted	Effective dose rate constant (µSv・m^2^・MBq^−1^・h^−1^)
6.647 days	β^−^	0.176–12.2% 0.385–9.1% 0.498–78.6% Other	0.113–6.4% 0.208–11.0% Other 0.0555–4.5% Hf-Kα 0.0637–1.2% Hf-Kβ	14.5% 0.73%	0.00517

Cited from Radioisotope Pocket Data Book (11th ed.), Japan Radioisotope Association, 2011

Lu-177 has a physical half-life of 6.647 days. Lu-177 emits β-rays that have a short range in soft tissue (average 0.23 mm, max. 1.7 mm) and γ-rays. The radionuclide is produced by the Lu-176(n,γ)Lu-177 reaction. Lu is a rare earth element with the atomic number 71 [12].

### In vivo dynamics of Lu and the labeled somatostatin analogue

#### In vivo dynamics of Lu

Data on the in vivo dynamics of lutetium in humans have not been presented. However, data on inorganic compounds containing lutetium in laboratory animals have revealed that lutetium collects in tissue and organs (60% in bone, 2% in the liver, and 0.5% in the kidneys). In addition, lutetium is reported to have a biological half-life of 3500 days in bone and the liver and 10 days in the kidneys [13]. Thus, most of the lutetium taken up by the body collects in bone and it accumulates over time in that location.

#### In vivo dynamics of the labeled somatostatin analogue

Once a somatostatin analogue labeled with Lu-177 is intravenously administered to a patient with a neuroendocrine tumor, Lu-177 is quickly excreted in the urine. Based on the radiation in patient excreta, Wehrmann et al. estimated that residual radiation from the labeled somatostatin analogue in the body decreased to about 30% of the dose 24 h after administration and to about 20% 48 h after administration [14]. Sandström et al. studied in vivo dynamics in patients administered a labeled somatostatin analogue, and reported that Lu-177 had a half-life with two phases [15], as shown in Table 3.

**Table 3:** Effective half-life of Lu-177 in a patient administered the labeled somatostatin analogue

Time after admin.	Effective half-life
0–24 h (early phase)	1.28 h (range 0.93–1.52 h)
24–168 h (late phase)	49.5 h (range 45.1–56.6 h)

## Release of patients administered a radiopharmaceutical

Article 30, Section 15, Subsection 1 of the Ordinance for Enforcement of the Medical Services Act (admissions restrictions) stipulates that “the administrator of a hospital or clinic shall not admit a patient who is being treated with radiation-producing medical equipment or a radiation-producing medical device implanted or a patient who is being treated with a radiopharmaceutical or a positron emission tomography radiopharmaceutical to patient rooms other than rooms for radiation therapy.” [16]. Provisions of the Ordinance also seek to reduce the exposure of third parties, i.e., individuals other than the treated patient. That said, the same provisions “are not applicable when implementing appropriate safeguards and steps to prevent contamination.” When a certain level of radiation protection is provided, the QOL of the treated patient will be taken into account and the administrator of a hospital or clinic is not necessarily obligated to admit a treated patient to a room for patients undergoing radiation therapy. This is the import of the guidelines for the “Release of Patients who have been Administered a Radiopharmaceutical.”

### Release criteria for patients administered a radiopharmaceutical

Release criteria (Notice No. 70 from the Safety Division, Pharmaceutical and Medical Safety Bureau) have been laid out as guidelines to enhance the QOL of treated patients and to ensure the safety of the general public and caregivers from radiation. These criteria were publicized as an interpretation of the “provisos” stipulated in Article 30, Section 15, Subsection 1 of the Ordinance for Enforcement of the Medical Services Act. The gist of the release criteria is generally as follows:

1. *Scope* When patients who have been administered a radiopharmaceutical are released or discharged from rooms where radiopharmaceuticals are used or rooms for patients undergoing radiation therapy in a hospital or other medical facility.
2. *Release criteria* The “criteria for a reduced dose” stipulate that the dose for the general public will be 1 mSv each year. Given the benefit for both patients and caregivers, the dose for caregivers will be 5 mSv per course of treatment [17].
  In specific terms, a patient may be released or discharged when any of the following apply.
  1. Release criteria based on dose
    A patient may be released or discharged when the dose or level of residual radioactivity in the body does not exceed the level of radioactivity as stipulated in Table 4.
    **Table 4:** Level of radioactivity during the release or discharge of patients administered a radiopharmaceutical

Nuclides used in therapy	Dose or level of residual radioactivity in the body (MBq)
Strontium-89	200^a^
Iodine-131	500^b^
Yttrium-90	1184^a^

^a^Max. dose

^b^The radioactivity of Iodine-131 will be determined by combining the dose of external exposure from a patient’s body and the dose of internal exposure as a result of inhalation of iodine-131 expelled while the patient breathes
  2. Release criteria based on the measured dose rate
    A patient may be released or discharged when the dose rate measured at a point 1 m from the surface of the patient’s body does not exceed the value in Tables 5 and 6.
    **Table 5:** Dose rate for release or discharge of patients administered a radiopharmaceutical

Nuclide used in therapy	1-cm dose equivalent rate 1 m from the surface of the patient’s body (µSv/h)
Iodine-131	30^a^

^a^The dose equivalent rate will be determined by combining the dose of external exposure from a patient’s body and the dose of internal exposure as a result of inhalation of iodine-131 expelled while the patient breathes
    **Table 6:** Examples where release criteria based on evaluation of the cumulative dose per patient have been met

Nuclide used in therapy	Scope	Dose (MBq)
Iodine-131	Destruction of residual thyroid tissue (ablation) after a complete thyroidectomy to treat differentiated thyroid cancer with no distant metastasis^a^	1110^b^

^a^*Conditions for treatment* Treatment will only be performed in accordance with the treatment guide (Outpatient Treatment with I-131 (1110 MBq) to destroy residual thyroid tissue) created by relevant academic societies and associations

^b^The radioactivity of Iodine-131 will be determined by combining the dose of external exposure from a patient’s body and the dose of internal exposure as a result of inhalation of iodine-131 expelled while the patient breathes
  3. Release criteria based on calculation of the cumulative dose per patient
    A patient may be released or discharged in the following situations based on the cumulative dose calculated per patient (remainder omitted).
3. Records of release
  When a patient is released, the following items will be recorded and records will be retained for 2 years after release.
4. Dose, date/time of release, measured dose rate during release
5. Precautions and guidance for mothers of nursing infants
6. When a patient is released based on (3) under (2) above, the method used to calculate the cumulative dose for that release (remainder omitted)
7. Precautions
8. When a patient is released or discharged, the patient will be cautioned and instructed verbally and in writing with regard to his or her routine activities so as to avoid unnecessary exposure of third parties.
9. If a patient is nursing an infant, the patient will be fully informed, cautioned, and instructed.
10. Based on guidelines created by organizations such as academic societies and associations related to radiation, protection will be provided in accordance with the physical characteristics of the radionuclide, patients and caregivers will be informed, and other steps will be taken to ensure safety from radiation and to safely manage radiation.

### Factors related to the evaluation of release criteria

1. *Exposure factor* The duration of patient contact, the distance from the patient, and the radiation dose are elements of the dose from external exposure. Thus, the exposure factor is a factor that should be taken into account when evaluating the exposure dose to a third party. The exposure factor is determined by the extent of interaction with the patient.
2. Exposure factor for caregivers: 0.5
  When a patient requires extensive care, the exposure factor is, based on measurements of the exposure dose for patients administered a radiopharmaceutical, reasonably set at 0.5 according to one study [18]. A Japanese study that measured the exposure dose from patients who had been administered radiopharmaceuticals also indicated that an exposure factor of 0.5 was appropriate [19]. Thus, an exposure factor of 0.5 is used to evaluate the dose for caregivers after a patient is released or discharged.
3. Exposure factor for the general public: 0.25
  In a typical home, an exposure factor of 0.25 is appropriate based on one’s study’s measurements of the exposure dose for a patient’s family members [18]. An exposure factor of 0.25 is used for family members other than caregivers and members of the general public after a patient is released or discharged.

## Release of a patient administered the labeled somatostatin analogue

### Exposure dose for a third party from a patient administered the labeled somatostatin analogue

The exposure dose for third parties such as caregivers and the general public includes external exposure to radiation emitted by radioactive material in the body of a patient administered the labeled somatostatin analogue and internal exposure due to contamination from a patient’s excreta. The dose to which a third party is exposed is comprehensively evaluated as follows:

### Evaluation of the dose from external exposure

#### Effective dose rate of external exposure at a distance of 1 m from a patient administered the labeled somatostatin analogue

The formula for calculation of the dose rate for external exposure to a third party exposed to a patient administered the labeled somatostatin analogue.

5.2.1 $$I=A \times C \times {F_{\text{a}}} \div {L^2},$$

Here *I* effective dose rate [µSv/h] at a determined reference point, *A* residual radiation in the body [MBq] of a patient administered the labeled somatostatin analogue, *C* effective dose rate constant for Lu-177 [µSv m^2^ MBq^−1^ h^−1^]: the value 0.00517 [µSv m^2^ MBq^−1^ h^−1^] from Table 2 in “Characteristics of Lu-177” will be used. *F*_a_ effective dose transmission (when multiple shields are used, the sum of the transmission of each shield will serve as the total transmission), *L* distance [m] from a radiation source to a reference point.

#### Cumulative dose to which a third party is exposed from a patient administered the labeled somatostatin analogue

The formula for calculation of the cumulative effective dose when a third party is continuously exposed to radiation from a patient administered the labeled somatostatin analogue.

5.2.2 $$E=A \times \int_{0}^{\infty } {{{\left( {\frac{1}{2}} \right)}^{\frac{t}{T}}}{\text{d}}t} \times C \times {f_0},$$

Here *E* cumulative effective dose [µSv] to which a third party is exposed. *A* residual radiation in the body [MBq] of a patient administered the labeled somatostatin analogue. *C* effective dose rate constant for Lu-177 [µSv m^2^ MBq^−1^ h^−1^]. The value 0.00517 [µSv m^2^ MBq^− 1^ h^− 1^] from Table 2 in “Characteristics of Lu-177”. *T* physical half-life of Lu-177. *f*_0_ exposure factor (caregivers: 0.5; members of the general public besides caregivers: 0.25).

#### Factors for evaluation of the cumulative dose for caregivers and the general public from a patient administered the labeled somatostatin analogue

The cumulative dose to which a third party is exposed after a patient administered the labeled somatostatin analogue is released or discharged is calculated based on the effective dose rate at a distance of 1 m from the surface of the patient’s body.
Radiation in the body of a patient administered the labeled somatostatin analogue depends on the effective half-life of Lu-177, which involves both its physical half-life and in vivo dynamics of the labeled somatostatin analogue. After administration of the labeled somatostatin analogue, the cumulative dose for a third party is based on 2 aspects. One is the fact that residual radiation in the body decreases to about 30% of the dose 24 h after administration and to about 20% of the dose 48 h after administration. As was mentioned in 3.2.2, this finding is from a study by Wehrmann et al. [14] that estimated the radiation in excreta from patients administered a labeled somatostatin analogue. The second aspect is the fact that Lu-177 is metabolized in two phases [effective half-life in the early phase: 1.28 h (range: 0.93–1.52 h); effective half-life in the late phase: 49.5 h (range: 45.1–56.6 h)]. This finding is from a study by Sandström et al. [15] that examined Lu-177 metabolism after administration of a labeled somatostatin analogue.
1) and 2) summarize the factors used to provisionally calculate the cumulative dose to which a third party is exposed from a patient administered the labeled somatostatin analogue. These factors are used to provisionally calculate changes in Lu-177 radiation in the body.
Dose of the labeled somatostatin analogue: 7400 MBq
Effective half-life in a patient after administration of the labeled somatostatin analogue: early phase: 1.52 h, late phase: 56.6 h.
  Sandström et al. [15] reported effective half-lives of Lu-177. The effective half-life of Lu-177 in a patient administered the labeled somatostatin analogue is a conservative estimate based on these half-lives. The effective half-life during the first phase will be 1.52 h and that during the second phase 56.6 h.
Accumulation (%) of the labeled somatostatin analogue in the tumor and organs [14]: 30% of the dose.
Distribution (%) of the labeled somatostatin analogue in tissue and organs other than the tumor and affected organ [14]: 70% of the dose.

#### Provisional calculation of the cumulative dose of external exposure for a third party exposed to radiation from a patient administered the labeled somatostatin analogue

1. Estimation of the effective dose rate over time at a distance of 1 m from the surface of the patient’s body after administration of the labeled somatostatin analogue.
  Based on 3) in "Factors for evaluation of the cumulative dose for caregivers and the general public from a patient administered the labeled somatostatin analogue", the effective dose rate of external exposure at a distance of 1 m from the surface of the patient’s body can be determined at a certain point after administration of the labeled somatostatin analogue using the following formula.
  5.2.4 $${I_{\text{d}}}=7400\left[ {{\text{MBq}}} \right] \times \left( {{e^{ - \left( {\frac{{0.693}}{{\left( {{\raise0.7ex\hbox{${56.6}$} \!\mathord{\left/ {\vphantom {{56.6} {24}}}\right.\kern-0pt}\!\lower0.7ex\hbox{${24}$}}} \right)}} \times {\text{d}}} \right)}} \times 0.3+e{}^{{ - \left( {\frac{{0.693}}{{\left( {{\raise0.7ex\hbox{${1.52}$} \!\mathord{\left/ {\vphantom {{1.52} {24}}}\right.\kern-0pt}\!\lower0.7ex\hbox{${24}$}}} \right)}} \times {\text{d}}} \right)}} \times 0.7} \right) \times 0.00517\left[ {{\raise0.7ex\hbox{${\mu {\text{Sv}}}$} \!\mathord{\left/ {\vphantom {{\mu {\text{Sv}}} {\left( {{\text{MBq}} \times {\text{h}}} \right)}}}\right.\kern-0pt}\!\lower0.7ex\hbox{${\left( {{\text{MBq}} \times {\text{h}}} \right)}$}}} \right] \times 1,$$
  *I*_d_ effective dose rate [µSv/h] × d after administration
2. Effective dose rate at a distance of 1 m from the surface of the patient’s body 24 h after administration of the labeled somatostatin analogue
  $${I_{1{\text{h}}}}={\text{ }}\left( {8.56{\text{ }}+{\text{ }}4.74 \times {{10}^{ - 4}}} \right)={\text{ }}8.56{\text{ }}\left[ {\mu {\text{Sv}}/h} \right].$$
3. Effective dose rate at a distance of 1 m from the surface of the patient’s body 48 h after administration of the labeled somatostatin analogue
  $${I_{2{\text{h}}}}={\text{ }}\left( {6.38{\text{ }}+{\text{ }}8.39 \times {{10}^{ - 9}}} \right)={\text{ }}6.38{\text{ }}\left[ {\mu {\text{Sv}}/{\text{h}}} \right].$$
  The effective dose rate at a distance of 1 m from the surface of the patient’s body is calculated to be 8.56 [µSv/h] 24 h after administration of the labeled somatostatin analogue and 6.38 [µSv/h] 48 h after administration. These dose rates are close to the effective dose rates of 8.0 ± 3.0 [µSv/h] and 6.2 ± 1.7 [µSv/h] that Archer et al. [20] measured 24 and 48 h after administration. When the in vivo dynamics of all of the radiation from the labeled somatostatin analogue are assumed to change depending solely on the early phase (effective half-life: 1.52 h), the effective dose rate at a distance of 1 m 24 h after administration can be determined using the following formula.
  $${I_{1.52 - 1{\text{h}}}}=7400\left[ {{\text{MBq}}} \right] \times {e^{ - \left( {\frac{{0.693}}{{(1.52/24)}} \times 1} \right)}} \times 0.00517\left[ {\mu {\text{Sv}}/\left( {{\text{MBq}} \times {\text{h}}} \right)} \right]=6.77 \times {10^{ - 4}}\left[ {\mu {\text{Sv}}/{\text{h}}} \right].$$
  Compared to 8.56 [µSv/h] as was determined in (5.2.4.1) ①, 6.77 × 10^−4^ [µSv/h] is a markedly lower dose rate. Thus, the extent of the residual radiation 24 h after administration depends on the effective half-life in the late phase (56.6 h).
4. The conditions under which to calculate the cumulative dose for a third party exposed to radiation from a patient administered the labeled somatostatin analogue at a dose of 7400 MBq are as follows:
5. Estimation of the cumulative dose of external exposure for a third party immediately after administration of the labeled somatostatin analogue
  The effective dose rate immediately after administration of the labeled somatostatin analogue (*d* = 0) can be determined using Eq. 5.2.4:
  $${I_0}={\text{ }}11.48{\text{ }}+{\text{ }}26.78{\text{ }}={\text{ }}38.26{\text{ }}\left[ {\mu {\text{Sv}}/{\text{h}}} \right].$$
  The cumulative dose of external exposure will be calculated for caregivers and the general public when they come into contact with a patient immediately after, 24 h after, and 48 h after administration of the labeled somatostatin analogue (①–③).
6. The cumulative dose of external exposure for a third party at a distance of 1 m from the surface of the patient’s body immediately after administration
  (11.48 [μSv/h] × (2.36 [day]/0.693) + 26.78 [μSv/h] × (0.063 [day]/0.693)) × 24 [h/day] × 4 [rounds/course of treatment] ÷ 1000 [μSv/mSv] = 3.99 [mSv/course of treatment].
  Cumulative dose for caregivers (exposure factor: 0.5): 3.99 [mSv/course of treatment] × 0.5 = 2.00 [mSv/course of treatment].
  Cumulative dose for the general public (exposure factor: 0.25): 3.99 [mSv/course of treatment] × 0.25 = 1.00 [mSv/course of treatment].
7. The cumulative dose of external exposure for a caregiver or the general public from a patient 24 h after administration.
  (8.56 [μSv/h] × (2.36 [day]/0.693) + 4.74 × 10^−4^ [μSv/h] × (0.063 [day]/0.693)) × 24 [h/day] × 4 [rounds/course of treatment] ÷ 1000 [μSv/mSv] = 2.80 [mSv/course of treatment].
  Cumulative dose for a caregiver (exposure factor: 0.5): 2.80 [mSv/course of treatment] × 0.5 = 1.40 [mSv/course of treatment]
  Cumulative dose for the general public (exposure factor: 0.25): 2.80 [mSv/course of treatment] × 0.25 = 0.70 [mSv/course of treatment]
8. The cumulative dose of external exposure for a caregiver or the general public from a patient 48 h after administration.
  (6.38 [μSv/h] × (2.36 [day]/0.693) + 8.39x10^−9^ [μSv/h] × (0.063 [day]/0.693)) × 24 [h/day] × 4 [rounds/course of treatment] ÷ 1000 [μSv/mSv] = 2.08 [mSv/course of treatment].
  Cumulative dose for a caregiver (exposure factor: 0.5): 2.08 [mSv/course of treatment] × 0.5 = 1.04 [mSv/course of treatment]
  Cumulative dose for the general public (exposure factor: 0.25); 2.08 [mSv/course of treatment] × 0.25 = 0.52 [mSv/course of treatment]
  The cumulative dose to which a third party is exposed immediately after and at a certain point after a patient is administered the labeled somatostatin analogue at a dose of 7400 MBq was calculated. Results of those calculations are shown in Table 7.
  **Table 7:** Calculation of the cumulative dose to which a third party is exposed immediately after and at a certain point after a patient is administered the labeled somatostatin analogue at a dose of 7400 MBq

	Immediately after admin. (mSv/course of treatment)	24 h after admin. (mSv/course of treatment)	48 h after admin. (mSv/course of treatment)
Caregiver	2.00	1.40	1.04
General public	1.00	0.70	0.52
  As shown in Table 7, the cumulative dose of external exposure for a caregiver who is exposed immediately after a patient is administered the labeled somatostatin analogue would be 2 mSv. This figure is sufficiently lower than 5 mSv per course of treatment, which is the “criterion for a reduced dose” described in the release criteria. The cumulative dose of 1 mSv for the general public is the same value as the dose limit for the general public as recommended by the ICRP. Without considering the in vivo dynamics of the labeled somatostatin analogue and assuming that the cumulative dose decreases with the physical half-life of Lu-177 (6.647 days).
  38.26 [μSv/h] × (6.647 [day]/0.693) × 24 [h/day] × 4 [rounds/course of treatment] × 0.25 ÷ 1000 [μSv/mSv] = 8.81 [mSv/course of treatment].
  When a patient is released immediately after administration of the labeled somatostatin analogue (7400 MBq), the cumulative dose to which the general public is exposed is estimated to range from 1.00 to 8.81 [mSv/course of treatment]. In such an event, the cumulative dose might exceed 1 mSv per year.
  In this Manual, the effective dose rate at a distance of 1 m 24 h after administration of the labeled somatostatin analogue at a dose of 7400 MBq was provisionally calculated to be 8.56 [µSv/h]. Archer et al. [20] measured the effective dose rate at a distance of 1 m from patients 24 h after administration and reported that the effective dose rate was 8.0 ± 3.0 [µSv/h]. Thus, the cumulative dose to which an individual is exposed 24 h after a patient is administered the labeled somatostatin analogue was 11.0 [µSv/h] (8.0 + 3.0 [µSv/h]) according to Archer et al. [20]. Using this figure and the effective half-life of Lu-177 in a labeled somatostatin analogue [15] (56.6 h = 2.36 days), the integrated value of the dose from a patient 24 h after administration can be determined.
  11.0 [μSv/h] × (2.36 [day]/0.693) × 24 [h/day] × 4 [rounds/course of treatment] × 0.25 ÷ 1000 [μSv/mSv] = 0.90 [mSv/course of treatment].
  The cumulative dose to which the general public is exposed 24 h after a patient is administered the labeled somatostatin analogue at a dose of 7400 MBq would be less than 1 mSv per year, which is the dose limit for the general public as recommended by the ICRP.

### Evaluation of the dose from internal exposure

Excreta from a patient administered the labeled somatostatin analogue flows to a sewage treatment plant primarily as urine, which then flows into a river. After additional treatment, this liquid could be used as potable water. Thus, provisional calculation of the dose from internal exposure assumes that all of the radiation administered to a patient flows into a river. The Yodo River accepts a large quantity of treated effluent, so this water system will be used as a model to evaluate the dose from internal exposure.

- The average flow of the Yodo River water system is about 4.1 [TL] per year (annual average prior to 1991–1995).
- Population of the metropolitan Osaka area that obtains potable water from that water system: About 14.02 million (2012) (Osaka Prefecture + Nara Prefecture + Wakayama Prefecture + 1/2 of Hyogo Prefecture) [21].
- Total population of Japan: About 127.52 million (2012) [21].
- Population of the metropolitan Osaka area as a proportion of Japan’s total population: 10.99% (0.11).
- Number of patients with a gastroenteropancreatic neuroendocrine tumor in Japan: 11,642 (number of patients per 100,000 population: 2.69 with a pancreatic neuroendocrine tumor, 6.42 with a gastrointestinal neuroendocrine tumor) [22].
- Number of such patients with distant metastasis: 1176 (percent with distant metastasis: 19.9% with distant metastasis of a pancreatic endocrine tumor, 6.0% with distant metastasis of a gastrointestinal neuroendocrine tumor) [22].
  Assuming that Lu-177-DOTA-TATE will be administered to all of these patients.
- Number of patients in the metropolitan Osaka area eligible for treatment: 1176 × 0.11 = 129 (calculated with respect to the population).
  That said, Fig. 0.11 is with respect to the population of the metropolitan Osaka area. This is assuming that Lu-177-DOTA-TATE is administered to each patient at a dose of 7400 MBq in 4 rounds per year.
- Level of radioactivity of the total dose of Lu-177-DOTA-TATE administered to patients in the metropolitan Osaka area:
  7400 [MBq/administration] × 4 [rounds/patient] × 129 [patients] = 3.82 [TBq]
  Here, the entire quantity of Lu-177-DOTA-TATE is assumed to be discharged into the Yodo River water system, where it is entirely in a water-soluble form.
- Lu-177-DOTA-TATE concentration in the river:3.82 [TBq/] ÷ 4.1 [TL/y] = 0.93 [Bq/L].
  That said, 4.1 TL is the average annual flow of the Yodo River water system.
- Annual intake of Lu-177-DOTA-TATE per member of the general public (assuming 2 L of water for drinking per d) [23]:
  0.93 [Bq/L] × 2 [L/d] × 365 [d/y] = 678.90 [Bq/y]
- In the aforementioned instance, the dose from internal exposure in 1 y:
  678.90 [Bq/y] × 5.3 × 10^−7^ [mSv/Bq] = 0.36 [µSv/y].
  However, 5.3 × 10^−7^ [mSv/Bq] is the effective dose coefficient for ingestion of Lu-177 [24].
  0.36 µSv/year is substantially lower than 1 mSv, which is the annual dose limit for the general public. Even if the upper reaches of the Yodo River water system (e.g., Kyoto Prefecture) are contaminated to the same extent, Lu-177-DOTA-TATE would contribute less than 0.1% to the annual dose limit for the general public.

### Comprehensive evaluation of the doses from external and internal exposure

The doses from external (Table 7) and internal exposure (“Evaluation of the dose from internal exposure”) for caregivers and the general public were comprehensively evaluated when patients were administered the labeled somatostatin analogue at a dose of 7400 MBq (max. dose) in up to four rounds of treatment per year (as part of internal radiotherapy with the labeled somatostatin analogue) and released 24 h after administration of each round of treatment. The results of that evaluation are as follows:

$${\text{Caregivers }}1.40{\text{ }}\left[ {{\text{mSv}}} \right]{\text{ }}+{\text{ }}0.36{\text{ }}\left[ {\mu {\text{Sv}}} \right]{\text{ }}={\text{ }}1.40{\text{ }}\left[ {{\text{mSv}}} \right].$$

$${\text{General public }}0.70{\text{ }}\left[ {{\text{mSv}}} \right]{\text{ }}+{\text{ }}0.36{\text{ }}\left[ {\mu {\text{Sv}}} \right]{\text{ }}={\text{ }}0.70{\text{ }}\left[ {{\text{mSv}}} \right].$$

The exposure dose for caregivers was provisionally calculated to be 1.40 [mSv], and that for the general public 0.70 [mSv]. These doses meet the criteria for a reduced dose for both caregivers and the general public.

### Criteria for release of a patient administered the labeled somatostatin analogue from a room for patients undergoing the therapy

When a patient has been administered, the labeled somatostatin analogue (7400 MBq) to treat a neuroendocrine tumor, the conditions indicated in (1) and (2) below must be met for the patient to be released from a room for patients undergoing radiation therapy.

1. Over 24 h after administration of the labeled somatostatin analogue.
2. During release, a radiation-measuring device will be used to measure the 1-cm dose equivalent rate at a distance of 1 m from the surface of the patient’s body, and the 1-cm dose equivalent rate does not exceed 10 µSv/h.
3. In addition to 1) and 2), if any of the following circumstances exist in the home after discharge, admission of the patient to a room for patients undergoing radiation therapy 48 h after administration must be considered.
  If the patient lives with highly radiosensitive infants and children (under the age of 15) or pregnant women.
  If the patient is unable to sleep at least 2 m away from individuals in the same household (preferably in a separate room).
  If the patient is incontinent and he or she requires a diaper or urinary catheter.
  If the patient must use the same form of public transportation for 2 h or longer upon discharge.
4. A patient to whom the labeled somatostatin analogue has been administered should be admitted to a room for patients undergoing radiation therapy. Rooms for patients undergoing radiation therapy are stipulated in Article 30, Section 12 of the Ordinance for Enforcement of the Medical Services Act. In addition, rooms for patients to whom the labeled somatostatin analogue has been administered must be rooms where “appropriate safeguards and steps to prevent contamination” are taken as stipulated in Article 30, Section 15 of the Ordinance for Enforcement of the Medical Services Act. The administrator of a hospital or other medical facility must manage and utilize those rooms in accordance with the criteria in the addenda to this Manual on proper use of a labeled somatostatin analogue.
5. Records related to 1) to 4) will be kept in a format as specified by this Manual on proper use of a labeled somatostatin analogue or addenda and those records will be retained for a given period.

### Precautions for patients and their families

After administration of the labeled somatostatin analogue, minute levels of radiation may be present in bodily fluids (principally blood), urine, and feces. Most of the labeled somatostatin analogue that is not taken up by a tumor will be eliminated by the kidneys and urinary tract. It was reported that a relatively high level of radiation is detected in urine for up to 48 h after administration, and so the precautions described in “Precautions in the week following administration of the labeled somatostatin analogue (first week after administration of a labeled somatostatin analogue)” to “Radiation safety management for a patient wearing a diaper or using a urinary catheter” must be explained to patients and their family members (caregivers) in writing prior to administration, and their assent must be obtained.

#### Precautions in the week following administration of the labeled somatostatin analogue (1st week after administration of a labeled somatostatin analogue)

Precautions with regard to routine activities:

① If a patient loses blood, that blood will be wiped up with toilet paper and flushed down the toilet.
② When there is any potential for coming into contact with a patient’s urine or feces and when coming into contact with clothing contaminated by a patient’s urine or feces, disposable latex gloves will be worn.
③ When a patient’s bodily fluids such as blood come into contact with the hands or skin, the contaminated site will be immediately washed with soap.
④ Sexual intercourse is prohibited.
⑤ Individuals living with a patient should be separated from the patient to the extent possible. A distance of at least 1 m should be maintained. When together for a prolonged period, a distance of 2 m or more should be maintained. Contact with infants and pregnant women will be minimized.
⑥ Sleeping with someone else in the same bed will be avoided. A patient should sleep at least 2 m away. If possible, the patient should sleep in a separate room.
⑦ The patient will bathe last. After bathing, the bathtub will be cleaned and washed with a brush and cleaning agent.
⑧ Outings in public settings (e.g. public transportation, supermarket, shopping centers, movie theaters, restaurants, and sport venues) should be avoided to the extent possible.
  When traveling by public transportation, the patient should be separate from other travelers (a distance of 1 m or greater). Travel time in the same vehicle will be reduced so that the patient does not spend more than 6 h on the same form of public transportation. When traveling by taxi, the patient will sit as far away from the driver as possible and travel time with the same driver will be reduced.

Precautions with regard to handling laundry:

① Clothing worn by a patient administered the labeled somatostatin analogue will be washed separately from the clothing of other individuals, and not at the same time. In addition, bed linens and undergarments soiled with blood or urine will be prepared for washing.

Precautions with regard to urination, defecation, or vomiting:

① Male patients will urinate while seated.
② When feces or urine soil the toilet or floor, that material will be wiped up with toilet paper and flushed down the toilet.
③ A toilet will be flushed about two times after use.
④ Hands will be washed and cleaned with soap after urination or defecation.
⑤ Hands and skin that come into contact with a patient’s bodily fluids (e.g. blood), excreta, or vomitus will be cleaned and washed with soap.

#### Precautions in the 3 months after administration of the labeled somatostatin analogue (first 3 months after administration of a labeled somatostatin analogue)

Precautions with regard to routine activities:

① When a patient is using facilities that detect radiation to prevent terrorism overseas (national borders, airports, etc.), the patient will carry proof of treatment such as a medical certificate.

#### Precautions in the 6 months after administration of the labeled somatostatin analogue (first 6 months after administration of a labeled somatostatin analogue)

Precautions with regard to routine activities:

① Female patients will avoid becoming pregnant or nursing and male patients will use condoms.

#### Precautions for patients after administration of the labeled somatostatin analogue

After administration, the labeled somatostatin analogue will quickly be excreted in the urine, and the level of residual radioactivity in the body is reported to decrease to about 30% of the original dose within 24 h of administration and to about 20% of that dose within 48 of administration [14].

#### Radiation safety management for a patient wearing a diaper or using a urinary catheter

The following precautions must be taken by a patient who is wearing a diaper or using a urinary catheter soon after administration (in principle, 1 week).

When handling a diaper, urinary catheter, or urine collection bag, disposable gloves will be worn, i.e., the same precautions as are followed to prevent biohazards will be taken.

Precautions when wearing a diaper or using a urinary catheter (at home or in the hospital):

① Vinyl sheets should be used by an incontinent patient who is wearing a diaper.
② When a patient is still using a urinary catheter despite being released from a room for patients undergoing radiation therapy, the urine in a urine collection bag will be disposed of in the toilet and the toilet will be flushed twice. After handling, hands will be washed and cleaned.
③ A catheter or urine collection bag will be replaced prior to the discharge of an inpatient.

Precautions during disposal of a diaper or urinary catheter:

① A diaper worn by a patient at home will be placed in a plastic bag and the bag will be closed so that the contents do not leak. The bag will be disposed of as ordinary garbage.
② When disposing of infectious waste such as diapers at a hospital, refer to Handling the Diapers of Patients who have been Administered a Radiopharmaceutical (Guidelines for Medical Personnel Working in Nuclear Medicine) (March 2001, 1st ed., March 2004, 2nd ed.) [25].

## Laws, ordinances, rules, and regulations on the clinical use of the labeled somatostatin analogue

When using a pharmaceutical in treatment as stipulated in Article 2, Section 1 of the Pharmaceuticals and Medical Devices Law, applicable laws, ordinances, rules, and regulations to prevent radiation injuries are generally the following:

① Law on the Prevention of Radiation Injuries due to Radioisotopes: Nuclear Regulatory Authority [26]
② Medical Services Act [27] (Ordinance for Enforcement of the Medical Services Act [28]): Ministry of Health, Labour, and Welfare
③ Pharmaceuticals and Medical Devices Law: Ministry of Health, Labour, and Welfare
④ Medical Practitioners Act: Ministry of Health, Labour, and Welfare
⑤ Pharmacists Act: Ministry of Health, Labour, and Welfare
⑥ Radiologic Technologists Act: Ministry of Health, Labour, and Welfare
⑦ The Law on Clinical Laboratory Technologists: Ministry of Health, Labour, and Welfare
⑧ Industrial Safety and Health Act (Ordinance on Prevention of Injuries from Ionizing Radiation [29] (denoted here as the “Ionizing Radiation Ordinance”) and the Act on Measurement of Workplace Conditions): Ministry of Health, Labour, and Welfare
⑨ National Civil Service Act (Rules of the National Personnel Authority 10-5 [30]): National Personnel Authority

### Criteria for rooms where radiopharmaceuticals are used

When a hospital or other medical facility performs treatment with a radiopharmaceutical like the labeled somatostatin analogue, the hospital or other medical facility must be equipped with rooms where radiopharmaceuticals are used, storage facilities, and disposal facilities that comply with criteria to prevent radiation injuries as stipulated in Article 30, Sections 8, 9, and 11 of the Ordinance for Enforcement of the Medical Services Act.

### Criteria for concentration limits in rooms where radiopharmaceuticals are used

A hospital or other medical facility performing nuclear medicine procedures must have facilities such as rooms where radiopharmaceuticals are used as indicated in “Criteria for rooms where radiopharmaceuticals are used”, and those facilities must comply with criteria for concentration limits as shown in Table 8.

**Table 8:** Criteria for dose limits and concentration limits in rooms where radiopharmaceuticals are used

Rooms where radiopharmaceuticals are used	Medical Services Act
Rooms where radiopharmaceuticals are used	Rooms where radiopharmaceuticals are used^a^
	Storage facilities^b^
	Disposal facilities^c^
	Rooms for patients undergoing radiation therapy^d^
Dose limits and concentration limits in controlled areas^e^	The effective dose of external radiation^f^: 1.3 mSv every 3 months Concentration of RIs in the air^f^: the average concentration over 3 months is 1/10 of the concentration limit of an RI in the air Surface density of a material contaminated with an RI^f^: 1/10 of the surface concentration limit (RIs that do not emit alpha rays: 4 Bq/cm^2^)
Dose limits and concentration limits at places in facilities using RIs where people are constantly entering^a,b,c^	The effective dose on painted walls: 1 mSv or less every week Concentration of an RI in the air^f^: the average concentration over 1 week is equal to the concentration limit of an RI in the air Surface density of a material contaminated with an RI^f^: surface concentration limit (RIs that do not emit alpha rays: 40 Bq/cm^2^)
Dose standards at boundaries in a hospital or other medical facility (including areas in the hospital where people stay)^g^	The effective dose is 250 µSv or less^f^ every 3 months
Exposure dose for inpatients^h^	The effective dose does not exceed 1.3 mSv every 3 months

^a^Article 30, Section 8 of the Ordinance for Enforcement of the Medical Services Act: rooms where radiopharmaceuticals are used

^b^Article 30, Section 9 of the Ordinance for Enforcement of the Medical Services Act: storage facilities

^c^Article 30, Section 11 of the Ordinance for Enforcement of the Medical Services Act: disposal facilities

^d^Article 30, Section 12 of the Ordinance for Enforcement of the Medical Services Act: rooms for patients undergoing radiation therapy

^e^Article 30, Section 16 of the Ordinance for Enforcement of the Medical Services Act: controlled areas

^f^Article 30, Section 26 of the Ordinance for Enforcement of the Medical Services Act: concentration limits

^g^Article 30, Section 17 of the Ordinance for Enforcement of the Medical Services Act: protection at property lines

^h^Article 30, Section 19 of the Ordinance for Enforcement of the Medical Services Act: preventing patient exposure

### Restrictions on places where radiopharmaceuticals can be used (Article 30, Section 12 of the Ordinance for Enforcement of the Medical Services Act)

A radiopharmaceutical must be handled in a room designed for radiopharmaceutical use. This provision does not apply when temporarily using a radiopharmaceutical in an operating room, when using a radiopharmaceutical in a room for patients undergoing radiation therapy since a patient cannot be readily moved, or when temporarily using a radiopharmaceutical in an intensive care unit or cardiac care unit where appropriate safeguards and steps to prevent contamination have been taken.

## Safety management at radiation facilities in conjunction with use of the labeled somatostatin analogue

### Management of radiation safety through usage records (Article 30, Section 23 of the Ordinance for Enforcement of the Medical Services Act)

When using the labeled somatostatin analogue, it must be used appropriately to protect individuals from radiation and must be stored in a specified location. Radiation safety must be managed by clearly indicating the location of the radioactive material. Accordingly, usage records in line with the following provisions will be kept and constantly managed [31].

#### Records of the receipt, use, storage, and disposal of the labeled somatostatin analogue (radiopharmaceutical usage records)

Article 30, Section 23, SubSection 2 of the Ordinance for Enforcement of the Medical Services Act, Guidance Document No. 6, 1974, Medical Affairs Bureau, Ministry of Health and Welfare, Notice No. 188 of the Pharmaceutical and Food Safety Bureau.

The following items are required on usage records.

① Product specifications, ② date of arrival, ③ date of use, ④ quantity used, ⑤ quantity remaining, ⑥ user, ⑦ name of patient, ⑧ date of storage or disposal, and ⑨ radiation levels during storage or disposal.

In addition, storage records for stored pharmaceuticals will be kept and the quantity stored at the facility will be periodically verified to ensure that the maximum quantity of each nuclide scheduled for storage is not exceeded.

#### Measurement and recording in places where radiation injuries could occur (Article 30, Section 22 of the Ordinance for Enforcement of the Medical Services Act, Article 54 of the ionizing radiation ordinance)

Radiation will be measured in rooms where RIs are used (painted wall rooms where RIs are used, rooms where RIs are used, storage rooms, and disposal facilities (waste collection rooms and drainage systems)), at boundaries of controlled areas, in living quarters, in rooms for patients undergoing radiation therapy, and on property lines. That measurement will be made once prior to the start of treatment and no later than 1 month after the start of treatment (a period not to exceed 6 months for specified locations). The radiation level and the extent of contamination from RIs will be measured and records of these results will be retained for 5 years. The 1-cm dose equivalent (rate) will be used to measure the level of radiation (if the 70-µm dose equivalent (rate) in a location is potentially ten or more times the 1-cm dose equivalent (rate), the 70-µm dose equivalent (rate) will be used). The level of radiation and extent of contamination from RIs will be measured with a radiation-measuring device. If measurement with a radiation-measuring device is extremely difficult, the level of radiation and extent of contamination from RIs can be calculated instead.

#### Records of the measurement and calculation of the exposure dose for radiology technologists and technicians (Article 30, Section 18 of the Ordinance for Enforcement of the medical Services Act, Article 8 of the ionizing radiation Ordinance)

The dose from external exposure and internal exposure will be measured. Based on those measurements, the effective dose and equivalent dose for radiology technologists and technicians will be calculated as stipulated by the Minister of Health, Labour, and Welfare (Notification No. 398 from the Ministry of Health and Welfare [24]).

#### Ionizing radiation medical Examination, personal form (Article 57 of the ionizing radiation ordinance)

Results of the Ionizing Radiation Medical Examination for personnel who regularly work in radiology (radiology technologists and technicians) will be recorded on the Ionizing Radiation Medical Examination, Personal Form.

### Records related to the release of a patient administered the labeled somatostatin analogue (Notice No. 70 from the safety Division, Pharmaceutical and medical safety Bureau, which was superseded by Notice No. 1108-2 from the division for guidance on medical care, Health Policy Bureau)

When a patient is released or discharged, the following items will be recorded and records will be retained for 2 years after release.

① Dose, date/time of release, measured dose rate during release
② Precautions and guidance for mothers of nursing infants

## Radiation measurement

### Dose (radiation) measurement

Like radioactive diagnostic agents such as Tc-99m and I-123 and radiopharmaceuticals such as Sr-89, Y-90, I-131, and Ra-223, the radioactivity of Lu-177 in a dose is measured with a well ionization chamber that is referred to as a dose calibrator (Curie meter). The method of measurement is the same as that used to measure the radioactivity of radioactive diagnostic agents. During measurement, Lu-177 in a specified sealed container (vial) and the well ionization chamber will be held in position with a jig. Lu-177 is a nuclide with no proven track record, and so there are instances in which a well-ionized chamber may not be calibrated to measure Lu-177 (the chamber does not have a calibration factor for Lu-177). When measuring the radioactivity of Lu-177 for the first time, the measuring device must be calibrated for Lu-177 beforehand or the manufacturer of the measuring device must be contacted to ascertain the calibration factor.

### Dose measurement in places where radiopharmaceuticals are used

When using a radiopharmaceutical, the air dose in controlled areas where people constantly enter, at boundaries of controlled areas, on property lines, and in living quarters must be measured periodically or as necessary. Otherwise, the radiation level during patient release or the personal dose for workers such as radiology technologists and technicians must be measured (“Measurement and recording in places where radiation injuries could occur (Article 30, Section 22 of the Ordinance for Enforcement of the Medical Services Act, Article 54 of the Ionizing Radiation Ordinance)”). To manage the radioactivity of Lu-177, the dose of gamma rays will be measured. The air dose in a location will be measured using a measuring device calibrated with the 1-cm dose equivalent ***H*** ^*^(10) to indicate the ambient dose. The exposure dose will be measured using a measuring device calibrated with the 1-cm dose equivalent *H*_p_ (10) to indicate the personal dose equivalent.

A survey meter is a device with an ionization chamber or a scintillation counter like a NaI(Tl) scintillation counter that can detect the air dose. An ionization chamber is suited to measurement in places with a relatively high dose rate, such as places where radiopharmaceuticals are used. A highly sensitive NaI(Tl) scintillation survey meter is effective at measuring low doses in places such as boundaries of controlled areas and property lines. To evaluate the cumulative dose over a given period like 1 week or 3 months, the instantaneous dose rate (typically expressed in µSv/h, though the instantaneous dose rate is actually the cumulative dose with a time constant of several seconds–several dozen seconds) is measured with the aforementioned survey meter. Based on the instantaneous dose rate, the cumulative dose during a given period can be appropriately calculated, but a measuring device that can measure the cumulative dose can also be used.

A personal dosimeter can be a dosimeter that directly displays the exposure dose or it can be a dosimeter that is worn for a certain period and then read with a reader that calculates the exposure dose (referred to as a passive dosimeter). A passive dosimeter is typically sent to a facility offering personal radiation monitoring to read the exposure dose. A dosimeter that directly displays the exposure dose is worn on the pocket, which is why it is also referred to as an instantly readable dosimetry badge. Many of the recent dosimeters contain a semiconductor like Si. Passive dosimeters are primarily film badges, though radiophotoluminescent glass dosimeters and optically stimulated luminescent dosimeters have recently been used.

## Training

### Training for a radiation safety supervisor when therapy with the labeled somatostatin analogue is performed (physicians or radiology technologists with sufficient knowledge of and experience with radiation therapy)

Knowledge regarding therapy with the labeled somatostatin analogue must be gained to ensure medical safety and safe handling of radioactive material. Thus, a radiation safety supervisor and a radiation safety officer will be present when performing the therapy. The radiation safety supervisor and radiation safety officer will participate in a workshop on radiation safety and handling for a given period. This workshop will be held at a hospital or other medical facility accredited by the Japanese Society of Nuclear Medicine. When conducted at a medical facility, training based on this Manual will include the following items:

① Laws and ordinances to prevent radiation injuries, item authorities must be notified of, and release criteria
② The chemical and physical properties of the labeled somatostatin analogue and protection from radiation
③ Instructions to prevent the exposure of medical personnel and patients and their families
④ Measurement of radioactivity and safe management of radioactive waste
  A physician with expertise gained through training conducted at a hospital can serve as the individual responsible for performing internal radiotherapy with the labeled somatostatin analogue. In such an event, the physician should be designated as such by the administrator of the hospital.
  Records of training conducted at the hospital will be kept. Training records will be retained for at least 2 years.

## Radiation protection for medical personnel and steps to prevent radioactive contamination

### Radiation safeguards with regard to handling the labeled somatostatin analogue

1. Provision of protective equipment
  ① *Protective eyewear (required)* Protective eyewear will be provided since the injectable could directly contaminate the eyes when the labeled somatostatin analogue is handled [caution must be exercised since internal radiotherapy with the labeled somatostatin analogue requires a massive dose of the labeled somatostatin analogue (7400 MBq/round of treatment)].
  ② *Wearing protective gloves (required)* To avoid direct contamination of the fingers when handling the labeled somatostatin analogue.
  ③ *Water-absorbent polyethylene-coated filter paper* Polyethylene-coated filter paper to absorb moisture containing radioactive material to avoid the spread of contamination. Polyethylene-coated filter paper will be placed where contamination might occur, such as inside a biological safety cabinet, on surrounding work surfaces, and on lead bricks.
  ④ *Forceps* Forceps with silicone-covered tips stop slippage and facilitate grasping of a vial with forceps.
  ⑤ *A tray of an appropriate size* A stainless steel tray of the appropriate size will be covered with water-absorbent polyethylene-coated filter paper, and the labeled somatostatin analogue will be dispensed over the tray. Even if radioactive liquid spills during handling, radioactive contamination will be limited to the tray, helping to prevent the spread of contamination.
2. Basics of handling radioactive material
  Attention must be paid to external exposure when handing a radiopharmaceutical, i.e., an unsealed RI. Attention must also be paid to internal exposure that may occur as a result of the radiopharmaceutical being taken up by the body. Unlike a sealed RI, a radiopharmaceutical is often handled at a short distance. After administration of the radiopharmaceutical, a patient must still be considered a potential source of radiation. When handling the labeled somatostatin analogue, the time procedures take should be reduced, the distance from the radiation source should be maintained, and shielding should be provided (the three principles of protection from external exposure). These steps can help reduce exposure.
  1. Conducting cold runs (practice handling the labeled somatostatin analogue)
    A cold run refers to performing the same procedures as when using an RI without actually using radioactive material (RIs). These procedures include use of vials and pipettors. ① Procedures can be ascertained and verified through repeating practice and mastery. ② Whether needed apparatus or protective items are available can be verified. ③ Radioactive material can be handled quickly and mistakes can be reduced. In other words, a radiation source is handled less (handling takes less time) and handing errors due to incorrect procedures can be reduced. The distance from the radiation source and the measured dose rate when shielding is not used are shown in Table 9.
    **Table 9:** Distance from a somatostatin analogue (labeled with Lu-177) and the measured dose rate

Distance from the surface of the vial	Dose rate (µSv/h/MBq)
1 m	0.00676
10 cm	0.541–0.676
On the surface	> 1.351

For a drug with a level of radioactivity per vial of 7400 MBq (date detected)
  2. Precautions for controlled areas
    Precautions for entering and exiting controlled areas and laboratories will be posted near entrances and exits in accordance with the provisions of the Medical Services Act. Radiology technologists and technicians who work with radiation must be fully informed of these precautions. Key precautions are as follows:
    ① An entry log will be kept.
    ② Radiology technologists and technicians will change into dedicated footwear for use in controlled areas, athletic shoes, or protective footwear.
    ③ Radiology technologists and technicians will change into dedicated work clothes for use in controlled areas.
    ④ A personal dosimeter such as a dosimetry badge will be worn on the chest by males and on the waist by females.
    ⑤ The fact that ventilation equipment in a ventilation system is in operation will be verified.
    ⑥ When handling a radiopharmaceutical, protective eyewear and protective gloves must be worn.
    ⑦ A radiopharmaceutical or radioactive material that is discarded after use will be immediately transferred to a waste collection room once procedures are complete.
    ⑧ Radioactive contamination will be tested for indoors after use of a radiopharmaceutical or radioactive material. If contamination is found, decontamination will immediately be performed.
    ⑨ Hands will be washed and cleaned with cleanser and running water.
    ⑩ The hands, feet, shirt cuffs, surface of clothing, footwear, etc. will be tested for contamination.
    ⑪ If no contamination is present, personnel may change footwear and clothing. If contamination is found, decontamination will be performed in accordance with instructions from a radiation officer.
    ⑫ An exit log will be kept.
    ⑬ Personal dosimeter readings will be recorded.
  3. Handling the labeled somatostatin analogue
    *Dispensing the labeled somatostatin analogue* In principle, the labeled somatostatin analogue will be dispensed in a biological safety cabinet. The biological safety cabinet will be checked to ensure that it functions normally. In addition, polyethylene-coated filter paper will be placed on the floor near the biological safety cabinet to facilitate decontamination, and work surfaces in the cabinet, the back panel, and sides will be covered with polyethylene-coated filter paper as an when necessary. When handling a radiopharmaceutical, shielding such as lead sheets or bricks will be used to reduce exposure to radiology technologists and technicians.
    *Administration of the labeled somatostatin analogue* The labeled somatostatin analogue will be prepared in a fluid volume of 10–100 ml, so that it can be safely administered intravenously without causing perivascular damage. Foreign guidance on internal radiotherapy with a labeled somatostatin analogue has stated that a labeled somatostatin analogue should be administered via an indwelling catheter over 10–30 min [32]. An infusion system will allow a distance to be maintained and shielding to be used, thus limiting exposure to radiology technologists and technicians.
    *Procedures for handling the labeled somatostatin analogue and treatment of waste after administration* Protective eyewear will be used when handling the labeled somatostatin analogue. In addition, protective equipment such as lab coats and gloves will be worn. The labeled somatostatin analogue will be handled over a stainless steel tray covered with water-absorbent polyethylene-coated filter paper. Contaminants will be treated similarly. If the epidermis of the face or eyes is contaminated with the labeled somatostatin analogue, the site will immediately be cleaned with cleanser and running water.
    When radiology technologists and technicians are working with radiation, such as when they are preparing radiopharmaceuticals, other personnel should leave the area or avoid walking through the area. Once procedures are finished, waste will immediately be separated and stored until disposal.
    *Testing for contamination and decontamination in rooms where the labeled somatostatin analogue is used (walls and floor)* A radiation-measuring device will be used to test the inside of biological safety cabinets and the floor for contamination with the labeled somatostatin analogue in accordance with the manner in which the labeled somatostatin analogue is used.
    Lu-177 emits beta rays and gamma rays, and so a radiation-measuring device that effectively and efficiently measures Lu-177 levels must be used to detect surface contamination. Preparation and dispensing other radiopharmaceuticals containing nuclides at the same time in a room where radiopharmaceuticals are used can lead to incorrect administration, and thus other radiopharmaceuticals should not be prepared and dispensed at the same time to ensure medical safety.
    A measuring device used to measure sites contaminated with Lu-177 can be used to separately measure beta rays and gamma rays with a high level of sensitivity. A survey with a Geiger-Mueller survey meter is the most effective way to test for contamination on a workbench or the floor.
    When radioactive contamination is found on a workbench or the floor, decontamination must be quickly performed. If contamination is found relatively early, the contaminant will be wiped up with paper towels and the site will be gradually decontaminated using water, a neutral detergent, or a chelating agent such as citric acid. These procedures are typical. Attention will be paid to tears or pin holes in the gloves that are used in decontamination to avoid secondary contamination of the body. If the site cannot be completely decontaminated, the extent of contamination, measurements, and the date of contamination will be clearly indicated with a permanent marker. Barriers preventing access to avoid the spread of contamination are an appropriate step to prevent exposure to radiation and to prevent contamination.

### Exposure to medical personnel (external exposure and internal exposure)

Based on Article 30, Sections 18 and 27 of the Ordinance for Enforcement of the Medical Services Act, Sections 2–5, Subsections 1 and 2 (Provisions regarding limits) and Sections 2–6, Subsections 1–5 (Calculation of doses) of the Notice No. 188 of the Pharmaceutical and Food Safety Bureau, medical facilities must strive to prevent the exposure of radiology technologists, technicians, and other medical personnel. The dose of the labeled somatostatin analogue is usually 7400 MBq, though that dose can be reduced depending on the patient’s liver and kidney function, the size of foci, and the number of metastases. The time procedures take the distance from the radiation source, and the dose from external exposure for medical personnel have been conservatively calculated using a dose of 7400 MBq (in phase III clinical trials conducted in Europe and the US, the dose was 7400 MBq per round of treatment [6]). These calculations are shown in Table 10 (dose from external exposure to medical personnel). The effective dose rate constant used in dose evaluation was 0.00517 [µSv m^2^ MBq^−1^ h^−1^] from Table 2. In accordance with “Radiation safeguards with regard to handling the labeled somatostatin analogue”, safeguards must be taken to reduce the dose from external exposure.

**Table 10:** Dose from external exposure to medical personnel

Stage	Effective dose (per person)			Skin dose (per person)			Dose limits	
	Time procedures take (min)	Distance (cm)	Exposure dose (mSv)	Time procedures take (min)	Distance (cm)	Exposure dose (mSv)	Effective dose limit (whole body)	Equivalent dose limit (skin)
Preparations	5	50	0.013	5	10	0.319	Radiology technologists and technicians: 50 mSv/year 100 mSv/5 years Women who may be pregnant 5 mSv/3 months	500 mSv/year
Administration	30	150	0.009	30	100	0.019		

The effective dose (mSv/w) *E* of internal exposure per week for personnel will be calculated using the formula below based on Notification No. 398 from the Ministry of Health and Welfare dated December 26, 2000 [24] (see the Manual on Management of Medical Radiation [33]).

$$E=e \times I,$$

Here *I* is the quantity of a radiopharmaceutical (Bq) inhaled in 1 week,

$$I={\text{ }}1.2{\text{ }} \times {\text{ }}{10^6} \times C \times t,$$

1.2 × 10^6^: air intake (cm^3^/h) of an adult in 1 h. *C* average level of radioactivity in air (Bq/cm^3^) per week. *t* time procedures take/week.

C = *A* × dispersal rate × number of days the labeled somatostatin analogue will be used in 1 week/(*V* × 10^6^ × 8 (h) × number of days the ventilation system is in operation in 1 week),*A* maximum quantity (Bq) of the labeled somatostatin analogue that will be used in 1 day. *V* indoor ventilation (m^3^/h). Ventilation *V* (m^3^/h) when the system is in operation 8 h/day.

When using the labeled somatostatin analogue, *A* is 7400 MBq, the dispersal rate is 0.001, the indoor ventilation in 1 day is 560 (m^3^/h) × 8 (h), the labeled somatostatin analogue will be used 1 day/week (number of days the labeled somatostatin analogue will be used), the ventilation system will be in operation 5 day/week, procedures will take 5 min (0.083 h), and *e* (effective dose coefficient when Lu-177 is inhaled) will be 1.0 × 10^−6^ (mSv/Bq). The effective dose *E* (mSv) as a result of internal exposure per week will be as follows:

$$C={\text{ }}7400{\text{ }} \times {\text{ }}{10^6} \times {\text{ }}0.001{\text{ }} \times {\text{ }}1/\left( {560{\text{ }} \times {\text{ }}{{10}^6} \times {\text{ }}8{\text{ }} \times {\text{ }}5} \right){\text{ }}={\text{ }}3.30{\text{ }} \times {\text{ }}{10^{ - 4}}\left( {{\text{Bq}}/{\text{c}}{{\text{m}}^3}} \right).$$

$$I={\text{ }}1.2{\text{ }} \times {\text{ }}{10^6} \times C \times {\text{ }}0.083 \times 1{\text{ }}={\text{ }}32.87{\text{ }}\left( {{\text{Bq}}} \right).$$

$$E=e \times I={\text{ }}1.0{\text{ }} \times {\text{ }}{10^{ - 6}} \times {\text{ }}32.87{\text{ }}={\text{ }}3.29{\text{ }} \times {\text{ }}{10^{ - 5}}\left( {{\text{mSv}}} \right).$$

### Precautions for medical personnel

Medical personnel performing internal radiotherapy with the labeled somatostatin analogue will fully understand this Manual and the in vivo dynamics of the labeled somatostatin analogue. Medical personnel will plainly explain the principles of protection from radiation as were mentioned earlier to a patient and his or her family members. In addition, a physician with expertise in performing internal radiotherapy with the labeled somatostatin analogue will conduct appropriate training for medical personnel and endeavor to establish a system for cooperation within the medical facility. When emergency treatment is required to save the life of a patient, appropriate treatment may take precedence over compliance with the aforementioned provisions on radiation protection.

When caring for a patient, attention will be paid to the following points in the week after administration.

1. When there is a potential for coming into contact with a patient’s urine, feces, or blood and when handling clothing contaminated by a patient’s urine, feces, or blood, gloves that are impermeable to water will be worn.
2. When medical personnel come into contact with a patient’s excreta or blood, the individual will immediately cleanse and fully wash contaminated sites such as hands and skin with soap.
3. Clothing contaminated with a patient’s excreta or blood will be washed separately from the clothing of others.

## Disposal of radioactive contaminants from medical sources (material contaminated with Lu-177)

Material contaminated with the labeled somatostatin analogue corresponds to “radioactive contaminants from medical sources” as stipulated in Article 30, Section 11 of the Ordinance for Enforcement of the Medical Services Act. Radioactive contaminants from medical sources will be stored and disposed of at “disposal facilities (storage and waste collection facilities)” in a hospital or other medical facility based on the provisions of Article 30, Section 11 of the Ordinance. In addition, a designated individual commissioned to dispose off material contaminated with a radiopharmaceutical or RI as described in Article 30, Section 14-2, Subsection 1 will be asked about the contaminants.

A diaper or urine collection bag soiled with human excreta or blood will be handled in accordance with Handling the Diapers of Patients who have been Administered a Radiopharmaceutical (Guidelines for Medical Personnel Working in Nuclear Medicine) and the Manual on Handling the Diapers of Patients who have been Administered a Radiopharmaceutical (Japanese Society of Nuclear Medicine, Japan Radiological Society, Japan Society of Radiological Technology, Japanese Society of Nuclear Medicine Technology, Japan Association on Radiological Protection in Medicine) [25].

## References

[1] Release of Patients who have been Administered a Radiopharmaceutical (Notice No. 1108–2 from the Division for Guidance on Medical Care, Health Policy Bureau dated November 8, 2010, Notice from the Head of the Division for Guidance on Medical Care, Health Policy Bureau, Ministry of Health, Labor, and Welfare).

[2] Release of Patients who have been Administered a Radiopharmaceutical (Notice No. 70 from the Safety Division, Pharmaceutical and Medical Safety Bureau dated June 30, 1998, Notice from the Head of the Safety Division, Pharmaceutical and Medical Safety Bureau.

[3] Swärd C, Bernhardt P, Ahlman H, Wängberg B, Forssell-Aronsson E, Larsson M (2010). [^177^Lu-DOTA^0^-Tyr^3^]-octreotate treatment in patients with disseminated gastroenteropancreatic neuroendocrine tumors: The value of measuring absorbed dose to the kidney. World J Surg.

[4] Kwekkeboom D, de Herder W, Kam B, van Eijck C, van Essen M, Kooij P (2008). Treatment with the radiolabeled somatostatin analog [^177^Lu-DOTA^0^,Tyr^3^]octreotate: toxicity, efficacy, and survival. J Clin Oncol.

[5] Bodei L, Cremonesi M, Grana CM, Fazio N, Iodice S, Baio SM (2011). Peptide receptor radionuclide therapy with ^177^Lu-DOTATATE: the IEO phase I–II study. Eur J Nucl Med Mol Imaging.

[6] ClinicalTrials.gov. A study comparing treatment with 177Lu-DOTA0-Tyr3-octreotate to octreotide LAR in patients with inoperable, progressive, somatostatin receptor positive midgut carcinoid tumours (NETTER-1). http://www.clinicaltrials.gov/ct2/show/NCT01578239?term=177Lu&rank=4.

[7] ICRP Publication 53. Radiation dose to patients from radiopharmaceuticals. Ann ICRP. 1988;18(1–4). 10.1016/s0146-6453(99)00006-8 10840563

[8] ICRP Publication 60. Recommendations of the International Commission on Radiological Protection. Ann ICRP. 1991;21(1–3). 2053748

[9] ICRP Publication 73. Radiological protection and safety in medicine. Ann ICRP. 1996;26(2). 8911634

[10] ICRP Publication 94. Release of patients after therapy with unsealed radionuclides. Ann ICRP. 2004;34(2). 10.1016/j.icrp.2004.08.001 15571759

[11] International basic safety standards for protection against ionizing radiation and for the safety of radiation sources, IAEA safety series, no. 115, 1996.

[12] Handbook of chemistry: pure chemistry. 5th ed. Chemical Society of Japan; 2004.

[13] ICRP Publication 30 (Part 3). Limits for intakes of radionuclides by workers. Ann ICRP. 1981;6(2–3). 7332161

[14] Wehrmann C, Senftleben S, Zachert C, Müller D, Baum RP (2007). Results of individual patient dosimetry in peptide receptor radionuclide therapy with 177Lu DOTA-TATE and 177Lu DOTA-NOC. Cancer Biother Radiopharm.

[15] Sandström M, Garske-Román U, Granberg D, Johansson S, Widstrom C, Eriksson B (2013). Individualized dosimetry of kidney and bone marrow in patients undergoing ^177^Lu-DOTA-octreotate treatment. J Nucl Med.

[16] Enforcement of a Ministerial Ordinance partially amending the Ordinance for Enforcement of the Medical Services Act (Notice No. 188 of the Pharmaceutical and Food Safety Bureau dated March 12, 2001, Notice from the Director General of the Pharmaceutical and Food Safety Bureau, Ministry of Health, Labor, and Welfare).

[17] ICRP Publication 103. The 2007 recommendations of the international commission on radiological protection. Ann ICRP. 2007;37(2–4). 10.1016/j.icrp.2007.10.003 18082557

[18] Release of Patients who have been Administered a Radiopharmaceutical (Office Memorandum dated June 30, 1998, Safety Division, Pharmaceutical and Medical Safety Bureau, Ministry of Health and Welfare). http://www.jrias.or.jp/statute/pdf/19980630_zimu_kanjya.pdf.

[19] Koshida K, Koga S (1989). Criteria for discharge of patients undergoing ^131^I treatment and criteria for release to regular patient rooms based on the dose of external exposure. Nucl Med.

[20] Archer J, Carroll M, Vinjamuri S (2013). Clearance of ^177^Lu-DOTATATE from patients receiving peptide receptor radionuclide therapy. RAD Mag.

[21] 2014 Statistics on Japan, Statistics Bureau, Ministry of Internal Affairs and Communications. 2014.

[22] Ito T, Igarashi H, Nakamura K, Sasano H, Okusaka T, Takano K (2015). Epidemiological trends of pancreatic and gastrointestinal neuroendocrine tumors in Japan: a nationwide survey analysis. J Gastroenterol.

[23] Guidelines for drinking-water quality. Vol. I. Recommendations, WHO. 2008.

[24] Methods of measuring radiation doses radiology technologists and technicians are exposed to and methods of calculating the effective dose and equivalent dose (Notification No. 398 from the Ministry of Health and Welfare dated December 26, 2000).

[25] Handling the diapers of patients who have been administered a radiopharmaceutical (guidelines for medical personnel working in nuclear medicine) (March 2001, 1st ed., March 2004, 2nd ed.), Japanese Society of Nuclear Medicine, Japan Radiological Society, Japan Society of Radiological Technology, Japanese Society of Nuclear Medicine Technology, Japan Association on Radiological Protection in Medicine. http://www.jsnm.org/files/paper/kaku/41-2/k-41-2-11.pdf.

[26] Law on the Prevention of Radiation Injuries Due to Radioisotopes (law no. 167 dated June 10, 1957).

[27] Medical Services Act (law no. 205 dated July 30, 1948).

[28] Ordinance for Enforcement of the Medical Services Act (Ministry of Health and Welfare Ordinance No. 50 dated November 5, 1948).

[29] Ordinance on Prevention of Ionizing Radiation Injuries (Ministry of Labor Ordinance No 41 dated September 30, 1972).

[30] Rule 10-5 of the National Personnel Authority (prevention of radiation injuries to personnel) (rule 10-5 of the National Personnel Authority dated September 25, 1963).

[31] Guidelines on the Management of Radioisotope Concentrations in Emissions and Effluent, Japan Radiological Society, Japan Society of Radiological Technology, Japanese Society of Nuclear Medicine, Japanese Society of Nuclear Medicine Technology, April 2001. http://www.jrias.or.jp/pet/pdf/haisui_haiki_guideline.pdf.

[32] John J, Zaknun L, Bodei J, Mueller-Brand ME, Pavel RP, Baum D, Horsch (2013). The joint IAEA, EANM, and SNMMI practical guidance on peptide receptor radionuclide therapy (PRRNT) in neuroendocrine tumours. Eur J Nucl Med Mol Imaging.

[33] Manual on Management of Medical Radiation, Revised ed. Japan Radioisotope Association, 2004.

